# Effectiveness of tolvaptan monotherapy and low-dose furosemide/tolvaptan combination therapy for hepatoprotection and diuresis in a rat cirrhotic model

**DOI:** 10.3164/jcbn.16-122

**Published:** 2017-05-16

**Authors:** Norikazu Tanabe, Taro Takami, Koichi Fujisawa, Toshihiko Matsumoto, Naoki Yamamoto, Isao Sakaida

**Affiliations:** 1 Department of Gastroenterology and Hepatology, Yamaguchi University Graduate School of Medicine, 1-1-1 Minami Kogushi, Ube, Yamaguchi 755-8505, Japan; 2 Center of Research and Education for Regenerative Medicine, Yamaguchi University Graduate School of Medicine, 1-1-1 Minami Kogushi, Ube, Yamaguchi 755-8505, Japan; 3 Department of Oncology & Laboratory Medicine, Yamaguchi University Graduate School of Medicine, 1-1-1 Minami Kogushi, Ube, Yamaguchi 755-8505, Japan; 4 Yamaguchi University Health Administration Center, 1677-1 Yoshida, Yamaguchi, Yamaguchi 753-8511, Japan

**Keywords:** tolvaptan, furosemide, liver fibrosis, hepatoprotection, diuresis

## Abstract

Spironolactone and furosemide, which are used to treat ascites associated with decompensated cirrhosis, are ineffective in treating refractory ascites. Hence, combination therapy with tolvaptan, a vasopressin V2 receptor antagonist, has been approved in Japan. Tolvaptan monotherapy and combination therapy with furosemide inhibit fibrosis in cardiac remodeling; hence, we examined these therapies in a rat cirrhotic model, including their usefulness in inhibiting hepatic fibrosis. In the present study, we used a model of hepatic fibrosis induced by a choline-deficient l-amino-acid-defined diet + diethylnitrosamine. Rats were divided into a low-dose furosemide group (15 mg/kg/day), a high-dose furosemide group (100 mg/kg/day), a tolvaptan monotherapy group (10 mg/kg/day), a low-dose furosemide/tolvaptan combination therapy group, and a control group which received neither furosemide nor tolvaptan; we then assessed diuretic effects and hepatic fibrosis. The tolvaptan monotherapy group and the furosemide/tolvaptan combination therapy group demonstrated significantly higher urine volume than the control group and the low-dose furosemide group. In addition, tolvaptan monotherapy and low-dose furosemide/tolvaptan combination therapy were found to inhibit hepatic fibrosis and yield a hepatoprotective effect by an antioxidative mechanism. The results of the present study suggest that tolvaptan monotherapy and low-dose furosemide/tolvaptan combination therapy are highly effective for hepatoprotection and diuresis.

## Introduction

Cirrhosis patients frequently demonstrate hepatic edema as a clinical symptom of decreased liver function. Persistent subjective and objective symptoms associated with hepatic edema reduce quality of life.^(1)^ Ascites associated with decompensated cirrhosis have long been treated with drugs such as spironolactone and furosemide.^(2)^ However, there are cases of refractory ascites in which these drugs have often failed to yield appreciable therapeutic effects, even when used in combination.^(3)^ Tolvaptan, a vasopressin V2 receptor antagonist, can be used to treat hyponatremia, hyponatremia secondary to syndrome of inappropriate antidiuretic hormone secretion, and fluid retention resulting from heart failure.^(4)^ Previously, we reported that tolvaptan improves hepatic edema and ascites,^(5)^ and is approved in Japan for the treatment of refractory ascites in cirrhosis. Because tolvaptan exhibits a diuretic effect without excretion of sodium, it may be used in combination therapy to treat fluid retention in cirrhosis when existing diuretics fail to achieve a sufficient therapeutic effect.

A few studies in cardiovascular medicine have shown that in rat models of acute and chronic myocardial infarction, tolvaptan monotherapy and combination therapy with furosemide inhibit fibrosis in cardiac remodeling.^(6,7)^ Fascinatingly, tolvaptan has also been reported to inhibit fibrosis in myocardium, which does not have V2 receptors.^(8)^

The liver, like the myocardium, also does not have V2 receptors.^(8)^ Thus, we examined tolvaptan monotherapy and combination therapy with furosemide in a rat cirrhotic model for their usefulness in inhibiting hepatic fibrosis.

## Materials and Methods

### Animals

Animals were reared in accordance with the Yamaguchi University Graduate School of Medicine’s ethical guidelines on the use of animals. Six-week-old male Wistar rats (140–160 g) were purchased from Japan SLC (Shizuoka, Japan) and maintained in a room at an animal experiment facility at the Yamaguchi University Graduate School of Medicine under controlled temperature (25°C) and lighting (12-h light/12-h dark) conditions. The rats were fed a powdered choline-deficient l-amino-acid-defined (CDAA) diet (Dyets Inc., Bethlehem, PA; product numbers 518753, 518754) and injected intraperitoneally 10 mg/kg diethylnitrosamine (DEN) (Sigma N0756) weekly for 16 weeks.

### Drug administration

The entire experiment was conducted over a course of 16 weeks. The drugs used were furosemide (Nichi-Iko Pharmaceutical Co., Ltd., Toyama, Japan) and tolvaptan (Otsuka Pharmaceutical Co., Ltd., Tokyo, Japan). The rats were divided into five groups (*n* = 7 per group): a low-dose furosemide group (15 mg/kg/day; F15 group), a high-dose furosemide group (100 mg/kg/day; F100 group); a tolvaptan monotherapy group (10 mg/kg/day; T10 group), a low-dose furosemide + tolvaptan combination therapy group (F15 + T10; F15T10 group), and a control group in which neither drug was administered (C group). The drugs were mixed with the CDAA diet and administered continuously for 16 weeks. The rats were allowed ad libitum access to water. Urine volume in all groups was measured 4 h after drug administration.

### Blood measurements

To obtain serum samples, the rats were euthanized, and blood was collected from the celiac artery. We measured total serum protein, albumin, bilirubin, asparate aminotransferase (AST), alanine aminotransferase (ALT), LDH, creatinine, urea nitrogen, sodium, potassium, and chloride.

### Histological and immunohistochemical examinations

Sections of the right lobe of the liver (around 3 mm thick) were harvested from all rats, fixed in paraformaldehyde for 24 h, and embedded in paraffin. These sections were stained with Sirius red to assess hepatic fibrosis. Immunostaining was performed with anti-alpha-smooth muscle actin (α-SMA) antibody to identify activated stellate cells and 8-hydroxy-2'-deoxyguanosine (8-OHdG) antibody to assess oxidative stress; these assessments were conducted with the avidin-biotin-peroxidase technique as described below.^(9)^

Briefly, 3-µm-thick tissue sections were deparaffinized in xylene and rehydrated with alcohol and water. Antigens were retrieved by heating in a microwave oven 10 mM citrate buffer (pH 6.0) containing the tissue sections at 95°C for 6 min. Endogenous peroxidase was blocked with methanol solution containing 0.3% hydrogen peroxide at room temperature for 30 min. Nonspecific reactions were blocked with rabbit serum (Vector Laboratories, Burlingame, CA) for 20 min. Primary antibody reaction was performed on the sections overnight at 4°C in a moist chamber with rabbit polyclonal α-SMA antibody (1:50) (ab7817; Abcam, Cambridge, MA) and anti-8-OHdG antibody (1:10) (N45.1; JaICA, Shizuoka, Japan). The sections were then washed with phosphate-buffered saline (PBS) three times and reacted with biotinylated secondary antibody at room temperature for 1 h. Bound antibodies were detected with the avidin-biotin-peroxidase complex (ABC) technique (Vector Laboratories).

The Sirius red-positive, α-SMA-positive areas of the liver sections were visualized using a Keyence BIOREVO BZ9000 microscope (Osaka, Japan) and a Provis microscope (Olympus, Tokyo, Japan) equipped with a charge-coupled device (CCD) camera. Computer-assisted image analysis was performed with MetaMorph software (Universal Imaging Corporation, Downingtown, PA). The Sirius red-positive, α-SMA-positive areas were displayed as percentages of the total surface area of the specimens. Numbers of 8-OHdG-positive cells were counted in each section using a Keyence BIOREVO BZ9000 microscope.

### Real-time quantitative polymerase chain reaction (PCR)

Expression of α-SMA, collagen I, tumor growth factor beta1 (TGF-β1), and tissue inhibitor of metalloproteinase 1 (TIMP-1) mRNA was assessed with real-time quantitative PCR.

Listed below are the primers used. Rat α-SMA: sense (5'-AGAACACGGCATCATCACCAAC-3'), antisense (5'-GCACAGCCTGAATAGCCACATAC-3'); rat collagen I: sense (5'-AGCGGTGAAGAAGGAAAGAGAGG-3'), antisense (5'-CAATAGGACCAGAAGGACCAGCA-3'); rat TGF-β1: sense (5'-AGGTAACGCCAGGAATTGTTGCTA-3'), antisense (5'-TGCGCCTGCAGAGATTCAAG-3'); rat TIMP-1: sense (5'-CTGCAGGCAGTGATGTGCAA-3'), antisense (5'-ACAGGTTTCCGGTTCGCCTAC-3'); rat GAPDH: sense (5'-GGCACAGTCAAGGCTGAGAATG-3'), antisense (5'-ATGGTGGTGAAGACGCCAGTA-3').

### Assessment of the antioxidative effect of tolvaptan

Tolvaptan was dissolved in dimethyl sulfoxide (DMSO). HepaRG cells were purchased from KAC Co., Ltd. A collagen-coated 96-well plate was seeded with 1 × 10^4^ cells per well, and tolvaptan was added to the culture medium (Maintenance and Metabolism Medium) at concentrations of 0–80 µM. After 24 h, the culture medium was replaced, *tert*-butyl hydroperoxide (*tert*-BHP) was added at a concentration of 150 µM, and HepaRG cells were cultured under oxidative stress loading. Twenty-four hours later, MTS assays were performed using CellTiter 96 AQueous One Solution Reagent (Promega, Madison, WI).

### Statistical analysis

Differences were analyzed with the Student’s *t* test or analysis of variance (ANOVA) for biochemical and histological data. In addition, the log-rank test was used for survival analysis. Results are displayed as mean ± standard deviation, and *p*<0.05 was considered statistically significant.

## Results

### Effects on urine volume and survival rates

Urine volume at 4 h after drug administration was significantly higher in the four treatment groups than in the C group. In addition, compared to the F15 group, urinary output significantly increased in T10, F100, and F15T10 groups (*p*<0.01), which demonstrated equivalent diuretic effects (Table 1).

**Fig. 1:**
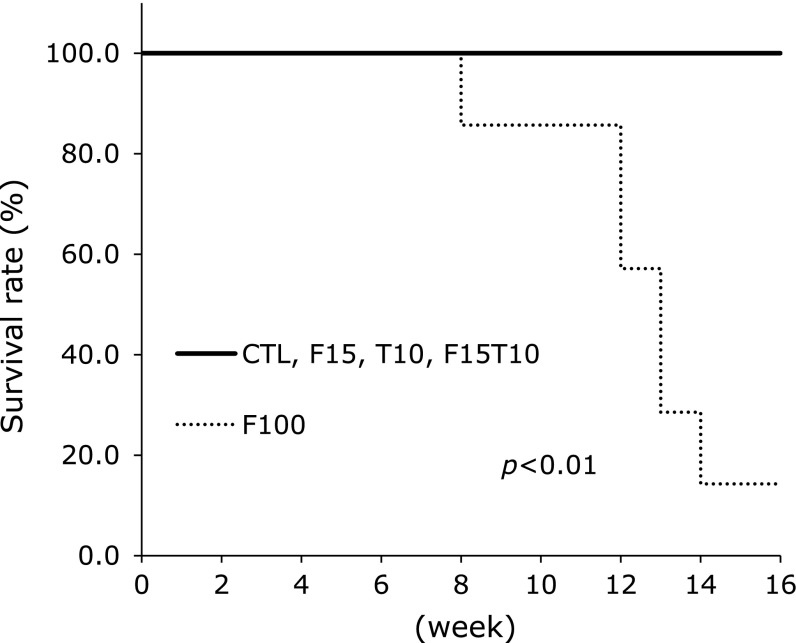
Survival curve analysis for each group. Log-rank analysis.

Fig. 1 shows the 16-week survival rates for each group. In the F100 group, 85.7% (6/7) of the rats died by the end of 16 weeks of diuretic administration; however, no rats died in the F15T10 group or in either of the other two treatment groups (*p*<0.01). For the F100 group, the large number of deaths made sample collection difficult. Due to the high death rate, the F100 group was eliminated from further investigations.

### Effects on metabolic parameters

The values of metabolic parameters at 16 weeks are shown in Table 1. Although body weight was roughly equal in all groups, liver weight significantly decreased in the treatment groups.

### Blood chemistry analysis

Table 1 shows serobiological makers of liver function at 16 weeks. No significant differences were observed in electrolyte levels. Compared to the C group, the tolvaptan groups (T10 and F15T10 groups) demonstrated a significant increase in albumin levels and a significant decrease in AST levels. The T10 group demonstrated a significant decrease in creatinine levels compared to the C group. In addition, the F15, T10, and F15T10 groups demonstrated a significant decrease in total bilirubin levels, as well as a tendency towards a decrease in ALT levels.

However, the F100 group demonstrated a marked increase in serum creatinine levels (1.30 ± 0.37 mg/dl) at 7 weeks; in the one rat that survived for 16 weeks, serum creatinine level was markedly high at 2.2 mg/dl.

### Histological and immunohistochemical examinations

**Fig. 2:**
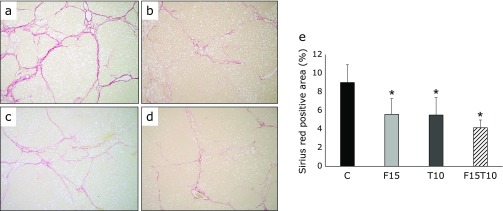
Histological analysis of hepatic fibrosis. Paraffin-embedded rat liver sections were stained with Sirius red (40× magnification). (a) CDAA + DEN only group, (b) CDAA + DEN + 15 mg/kg/day furosemide group, (c) CDAA + DEN + 10 mg/kg/day tolvaptan group, (d) CDAA + DEN + 15 mg/kg/day furosemide + 10 mg/kg/day tolvaptan group. (e) Image analysis of Sirius red-positive areas. Data are presented as mean ± SD. *p<0.01 vs CDAA + DEN group.

At 16 weeks, histological and immunohistochemical analyses of the liver revealed hepatic fibrosis in all groups. Compared to the C group, the Sirius red-positive area was significantly smaller in the treatment groups. No significant differences were observed among the treatment groups. Although the positive region was smaller in the F15T10 group, the difference was not statistically significant (Fig. 2).

**Fig. 3:**
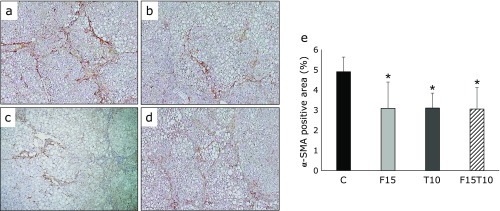
Immunohistochemical analysis of alpha-smooth muscle actin (α-SMA) expression in hepatic fibrosis. Paraffin-embedded rat liver sections were stained with α-SMA (40× magnification). (a) CDAA + DEN only group, (b) CDAA + DEN + 15 mg/kg/day furosemide group, (c) CDAA + DEN + 10 mg/kg/day tolvaptan group, (d) CDAA + DEN + 15 mg/kg/day furosemide + 10 mg/kg/day tolvaptan group. (e) Image analysis of α-SMA-positive areas. Data are presented as mean ± SD. *p<0.05 vs CDAA + DEN group.

Rats administered CDAA + DEN demonstrated marked proliferation of activated hepatic stellate cells, as observed by α-SMA staining. The treatment groups demonstrated significant reductions in α-SMA area compared to the control group. No significant differences were observed among the treatment groups (Fig. 3).

**Fig. 4:**
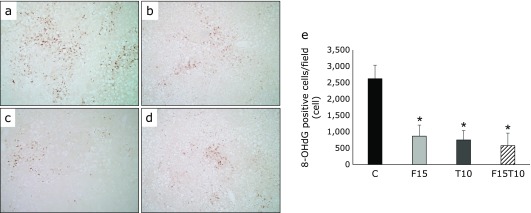
Immunohistochemical analysis of 8-hydroxy-2'-deoxyguanosine (8-OHdG) expression. Paraffin-embedded rat liver sections were immunostained with 8-OHdG (40× magnification). (a) CDAA + DEN only group, (b) CDAA + DEN + 15 mg/kg/day furosemide group, (c) CDAA + DEN + 10 mg/kg/day tolvaptan group, (d) CDAA + DEN + 15 mg/kg/day furosemide + 10 mg/kg/day tolvaptan group. (e) Image analysis of 8-OHdG-positive cells. Data are presented as mean ± SD. *p<0.01 vs CDAA + DEN group.

The number of cells positive for staining by 8-OHdG, a marker of oxidative DNA damage, was significantly lower in the treatment groups (Fig. 4).

### Effects on gene expression related to hepatic fibrosis

**Fig. 5:**
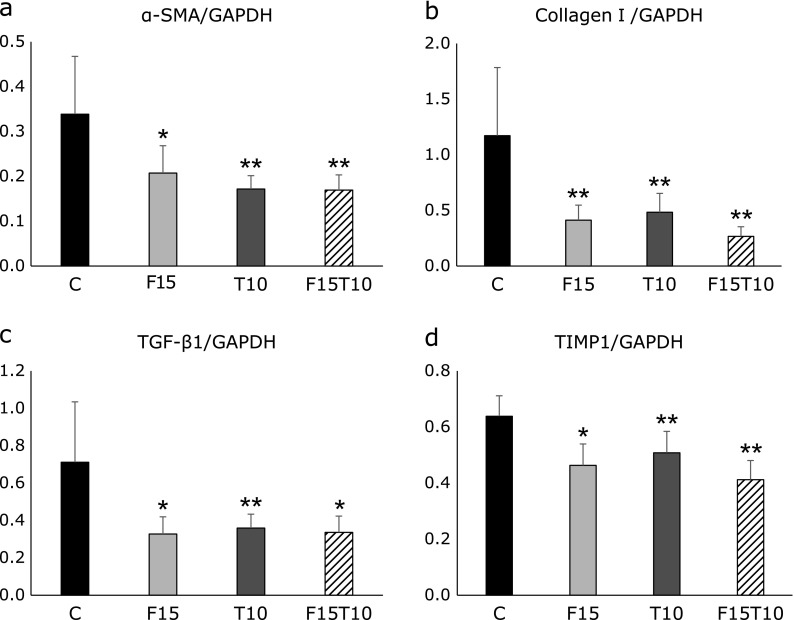
Real-time quantitative PCR analysis. (a) alpha-smooth muscle actin (α-SMA), (b) type I procollagen (Col I), (c) tumor growth factor beta (TGF-β1), (d) tissue inhibitor of metalloproteinase 1 (TIMP-1). Data are presented as mean ± SD. *p<0.05, **p<0.01 vs CDAA + DEN group.

We analyzed collagen I, TGF-β1, TIMP-1, and α-SMA mRNA expression in rat livers at 16 weeks. Expression of these transcripts was significantly inhibited in the treatment groups (Fig. 5).

### Antioxidative effect of tolvaptan

To assess the effect of tolvaptan on oxidative stress, we used HepaRG cells, which are reported to demonstrate human hepatocyte-like morphology and to retain and express the functions of human hepatocytes.^(10,11)^

**Fig. 6:**
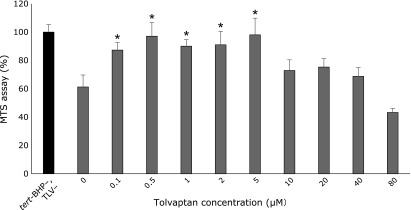
Tolvaptan demonstrated an antioxidative effect on tert-BHP-induced oxidative stress. MTS assay in HepaRG cells. Viability of HepaRG cells without drug administration was treated as 100%. Administration of *tert*-BHP alone caused the death of 40% of the cells. Tolvaptan was administered at concentrations of 0–80 µM. Data are presented as mean ± SD. *p<0.01 vs tolvaptan 0 µM.

Exposure of HepaRG cells to 150-µM *tert*-BHP for 24 h resulted in the death of approximately 40% of the cells. Compared to the administration of only *tert*-BHP, co-administration of 0.1–5 µM tolvaptan resulted in significant inhibition of cell death. No significant differences were observed with administration of ≥10 µM tolvaptan (Fig. 6).

## Discussion

Hepatic edema occurs when cirrhosis advances and becomes decompensated. Conventionally, hepatic edema has been treated with aldosterone drugs and loop diuretics; however, these sometimes fail to yield sufficient effects despite increase in dose or combined administration.^(3)^ In addition, higher doses and combined administration may upset the balance of electrolytes in the blood and impair renal function.^(12)^ For example, patients with cirrhotic ascites treated with furosemide have been reported to demonstrate significantly increased serum creatinine levels and BUN concentration, as well as significantly decreased glomerular filtration rate.^(13,14)^ Furthermore, by binding to albumin in blood, furosemide is secreted in the renal tubular lumen and is transported to the site of action, thereby resulting in insufficient action associated with hypoalbuminemia. Renal impairment and hyponatremia is associated with a poor prognosis for cirrhosis patients.^(15,16)^ Awareness has recently increased regarding the importance of renoprotection in cirrhosis.

Because tolvaptan exhibits a diuretic effect without causing excretion of sodium, it may be used in combination therapy to treat fluid retention in cirrhosis when existing diuretics fail to achieve a sufficient therapeutic effect.^(2)^ Tolvaptan exerts its effect irrespective of serum albumin levels; thus, significant reduction in body mass has also been observed in patients with hypoalbuminemia.^(17)^ Tolvaptan causes water diuresis leading to an increase in intravascular sodium levels and migration of extravascular water into the blood vessels, thereby retaining water in the blood vessels; this increases renal blood flow and prevents renal impairment. Long-term tolvaptan therapy in rat models of end-stage heart failure has been reported to improve kidney function, glomerular sclerosis, and interstitial fibrosis associated with oxidative stress.^(18)^

Hence, we examined the usefulness of tolvaptan monotherapy and combination therapy with furosemide in livers, which have no V2 receptors,^(8)^ of a rat cirrhotic model.

In the present study, we fed rats a CDAA diet, which triggered reactive oxygen species-related hepatocellular injury leading to hepatic fibrosis.^(19)^ Oxidative stress has also been demonstrated to play a major role in the progression of hepatitis C and nonalcoholic steatohepatitis. In hepatitis C, production of reactive oxygen species is reported to be enhanced.^(20)^ In nonalcoholic steatohepatitis, oxidative stress is reported to trigger production of inflammatory cytokines, which cause inflammation and fibrosis reaction.^(21)^

Tolvaptan has been reported to inhibit fibrosis in ventricular remodeling in rat models of acute and chronic myocardial infarction.^(6,7)^ Thus, tolvaptan has been shown to inhibit fibrosis in myocardium, which does not have V2 receptors. In addition, tolvaptan has been demonstrated to exert anti-inflammatory action and inhibit expression of TGF-β1 and collagen I mRNA. In the present study, low-dose furosemide, tolvaptan, and a combination of the two were found to reduce expression of α-SMA, collagen I, TGF-β1, and TIMP-1 mRNA; to reduce α-SMA-positive activated hepatic stellate cells; and to inhibit fibrosis in the liver. In blood chemistry analysis, tolvaptan monotherapy and low-dose furosemide/tolvaptan combination therapy were found to significantly increase albumin, significantly reduce AST, and yield a hepatoprotective effect. In addition, these therapies and low-dose furosemide monotherapy were found to result in a tendency towards reduced ALT and total bilirubin. The significant decrease in liver mass did not cause any difference in hepatic fat mass (data not shown) and was thus considered to reflect a hepatoprotective effect against hepatic dysfunction caused by CDAA diet + DEN.

In addition, tolvaptan monotherapy, low-dose furosemide monotherapy, and low-dose furosemide/tolvaptan combination therapy were found to reduce numbers of 8-OHdG positive cells, which reflect the degree of DNA oxidative stress in the liver. Treatment of HepaRG cells with tolvaptan led to an antioxidative effect under oxidative stress induced by tert-BHP. The antioxidative effect may be the mechanism by which tolvaptan exerts its hepatoprotective effects. To our knowledge, the present study is the first to assess the antioxidative effect of tolvaptan on hepatocytes.

An increase in serum renin activity triggered by furosemide results in the activation of the renin-angiotensin-aldosterone system,^(22)^ which may paradoxically enhance hepatic fibrosis.^(23)^ However, 10 mg/kg/day furosemide does not increase serum renin activity;^(22)^ thus, the low dose of furosemide used in the present study may have been insufficient to activate the renin-angiotensin-aldosterone system. In addition, furosemide is reported to exert an antioxidative effect and a hepatoprotective effect, which were obtained with low-dose furosemide.^(24,25)^

Tolvaptan is also used in clinical settings to treat autosomal dominant polycystic kidney disease. Although our study demonstrated that tolvaptan exerts a hepatoprotective effect, serious liver damage has also been reported. This potential for liver damage is reported by a study in which administration of ≥20 µM tolvaptan to HepG2 cells resulted in loss of viability.^(26)^ In the present study, an antioxidative effect was observed with tolvaptan at concentrations of 0.1–5 µM, but not with concentrations of ≥10 µM. In Japan, the upper limit of clinical administration of tolvaptan for refractory ascites in cirrhosis is 7.5 mg/day. In a pharmacokinetic study, the peak plasma concentration achieved after treatment with 7.5 mg/day tolvaptan was approximately 0.3 µM,^(27)^ which was within the range of tolvaptan concentrations that demonstrated an antioxidative effect in the present study. For autosomal dominant polycystic kidney disease, the upper limit of clinical administration is 60–120 mg/day; administration of 7.5 mg/day was suggested to pose little risk of liver damage and potentially yield a hepatoprotective effect.

Deterioration of kidney function was not observed in the low-dose furosemide monotherapy group, the tolvaptan monotherapy group, or the low-dose furosemide/tolvaptan combination therapy group in comparison to the control group. The tolvaptan monotherapy group demonstrated a significant decrease in serum creatinine; as reported in a previous study,^(17)^ tolvaptan may exert a renoprotective effect.

Urine volume, the fundamental purpose of administering diuretics, was significantly larger in the treatment groups than in the control group. In addition, urinary output was further higher in the T10, F100 and F15T10 groups than in the F15 group. However, high-dose furosemide was found to result in marked renal impairment from an early stage and lead to a high death rate. Although no kidney damage was observed in the F15 group, urine volume was insufficient. In the T10 and F15T10 groups, no kidney damage was observed, and unlike the F15 group, urine volume was sufficient.

In the present study, not only tolvaptan monotherapy and low-dose furosemide/tolvaptan combination therapy, but also low-dose furosemide monotherapy demonstrated equivalent effects in terms of hepatoprotection. However, low-dose furosemide monotherapy failed to yield sufficient urinary output.

Prior to the approval of tolvaptan, when treatment of fluid retention due to cirrhosis with spironolactone and furosemide failed to achieve appreciable therapeutic effects, the only recourse was to increase the doses. Currently, in Japan, tolvaptan is used in combination with existing diuretics. The results of the present study suggest that it is important to introduce tolvaptan from an early stage of low-dose furosemide administration than increase the dosage of furosemide. Although further investigation is necessary, the present study suggests that tolvaptan monotherapy may be highly useful.

## Figures and Tables

**Table 1:** Characteristics at the end of 16 weeks

	C (*n* = 7)	F15 (*n* = 7)	T10 (*n* = 7)	F15T10 (*n* = 7)	F100 (*n* = 7)
Body weight (g)	273.4 ± 7.7	267.5 ± 18.5	271.7 ± 18.5	273.0 ± 17.4	
Liver weight (g)	23.8 ± 0.3	19.5 ± 1.6**	20.1 ± 1.6**	19.1 ± 1.7**	
Urine volume (ml/kg/4 h)	4.5 ± 1.7	11.0 ± 2.2**	24.0 ± 3.2**	28.0 ± 6.2**	26.5 ± 5.9**
Serum					
T.Bil (mg/dl)	0.40 ± 0.11	0.23 ± 0.15**	0.28 ± 0.15**	0.25 ± 0.16**	
AST (IU/L)	389.8 ± 32.4	360.7 ± 57.9	335.0 ± 20.1**	293.7 ± 44.5**	
ALT (IU/L)	400.5 ± 68.9	383.1 ± 126.7	384.6 ± 26.2	335.1 ± 79.7	
LDH(IU/L)	2,103 ± 175	2,090 ± 777	1,793 ± 311	1,844 ± 713	
CRE (mg/dl)	0.33 ± 0.05	0.37 ± 0.07	0.27 ± 0.02*	0.36 ± 0.09	
BUN (mg/dl)	16.3 ± 3.7	15.9 ± 1.7	17.5 ± 2.3	20.4 ± 2.1*	
Na (mEq/L)	143.2 ± 0.7	143.9 ± 0.7	144.1 ± 1.5	143.7 ± 1.0	
K (mEq/L)	4.7 ± 0.4	4.8 ± 0.5	4.8 ± 0.5	4.5 ± 0.4	
Cl (mEq/L)	102.0 ± 1.8	102.7 ± 1.3	101.7 ± 1.1	101.6 ± 1.0	

Data are mean ± SD. *p<0.05 vs C group. **p<0.01 vs C group.

## Abbreviations

α-SMA: α-smooth muscle actin
ALT: alanine aminotransferase
AST: aspartate aminotransferase
CDAA: choline-deficient l-amino acid
DEN: diethylnitrosamine
DMSO: dimethyl sulfoxide
8-OHdG: 8-hydroxy-2'-deoxyguanosine
*tert*-BHP: *tert*-butyl hydroperoxide
TGF-β1: tumor growth factor beta1
TIMP-1: tissue inhibitor of metalloproteinase 1

## References

[1] Solà E, Watson H, Graupera I (2012). Factors related to quality of life in patients with cirrhosis and ascites: relevance of serum sodium concentration and leg edema. J Hepatol.

[2] Runyon BA (1997). Historical aspects of treatment of patients with cirrhosis and ascites. Semin Liver Dis.

[3] Wong F (2012). Management of ascites in cirrhosis. J Gastroenterol Hepatol.

[4] Zmily HD, Daifallah S, Ghali JK (2011). Tolvaptan, hyponatremia, and heart failure. Int J Nephrol Renovasc Dis.

[5] Sakaida I, Kawazoe S, Kajimura K (2014). Tolvaptan for improvement of hepatic edema: A phase 3, multicenter, randomized, double-blind, placebo-controlled trial. Hepatol Res.

[6] Yamazaki T, Izumi Y, Nakamura Y (2012). Tolvaptan improves left ventricular dysfunction after myocardial infarction in rats. Circ Heart Fail.

[7] Yamazaki T, Nakamura Y, Shiota M (2013). Tolvaptan attenuates left ventricular fibrosis after acute myocardial infarction in rats. J Pharmacol Sci.

[8] Ferguson JW, Therapondos G, Newby DE, Hayes PC (2003). Therapeutic role of vasopressin receptor antagonism in patients with liver cirrhosis. Clin Sci (Lond).

[9] Saeki I, Terai S, Fujisawa K (2013). Bortezomib induces tumor-specific cell death and growth inhibition in hepatocellular carcinoma and improves liver fibrosis. J Gastroenterol.

[10] Parent R, Marion MJ, Furio L, Trépo C, Petit MA (2004). Origin and charac-terization of a human bipotent liver progenitor cell line. Gastroenterology.

[11] Cerec V, Glaise D, Garnier D (2007). Transdifferentiation of hepatocyte-like cells from the human hepatoma HepaRG cell line through bipotent progenitor. Hepatology.

[12] Cárdenas A, Arroyo V (2005). Refractory ascites. Dig Dis.

[13] Bernardi M, De Palma R, Trevisani F, Santini C, Servadei D, Gasbarrini G (1985). Comparative pharmacodynamics of furosemide and muzolimine in cirrhosis. Study on renal sodium and potassium handling and renin-aldosterone axis. Z Kardiol.

[14] Ginés P, Arroyo V, Quintero E (1987). Comparison of paracentesis and diuretics in the treatment of cirrhotics with tense ascites. Results of a randomized study. Gastroenterology.

[15] Fede G, D'Amico G, Arvaniti V (2012). Renal failure and cirrhosis: a systematic review of mortality and prognosis. J Hepatol.

[16] Iwasa M, Ishihara T, Hasegawa H, Takei Y (2015). Cirrhosis-related hyponatremia and the role of tolvaptan. Hepatol Res.

[17] Sakaida I, Nakajima K, Okita K (2015). Can serum albumin level affect the pharmacological action of tolvaptan in patients with liver cirrhosis? A post hoc analysis of previous clinical trials in Japan. J Gastroenterol.

[18] Ishikawa M, Kobayashi N, Sugiyama F, Onoda S, Ishimitsu T (2013). Renoprotective effect of vasopressin v2 receptor antagonist tolvaptan in Dahl rats with end-stage heart failure. Int Heart J.

[19] Sakaida I, Hironaka K, Uchida K, Okita K (1999). Iron chelator deferoxamine reduces preneoplastic lesions in liver induced by choline-deficient L-amino acid-defined diet in rats. Dig Dis Sci.

[20] Valgimigli L, Valgimigli M, Gaiani S, Pedulli GF, Bolondi L (2000). Measurement of oxidative stress in human liver by EPR spin-probe technique. Free Radic Res.

[21] Rolo AP, Teodoro JS, Palmeira CM (2012). Role of oxidative stress in the pathogenesis of nonalcoholic steatohepatitis. Free Radic Biol Med.

[22] Hirano T, Yamamura Y, Nakamura S, Onogawa T, Mori T (2000). Effects of the V2-receptor antagonist OPC-41061 and the loop diuretic furosemide alone and in combination in rats. J Pharmacol Exp Ther.

[23] Yoshiji H, Kuriyama S, Yoshii J (2001). Angiotensin-II type 1 receptor interaction is a major regulator for liver fibrosis development in rats. Hepatology.

[24] Lahet JJ, Lenfant F, Courderot-Masuyer C (2003). *In vivo* and *in vitro* antioxidant properties of furosemide. Life Sci.

[25] Kang MY, Tsuchiya M, Packer L, Manabe M (1998). *In vitro* study on antioxidant potential of various drugs used in the perioperative period. Acta Anaesthesiol Scand.

[26] Wu Y, Beland FA, Chen S, Liu F, Guo L, Fang JL (2015). Mechanisms of tolvaptan-induced toxicity in HepG2 cells. Biochem Pharmacol.

[27] Sakaida I, Yanase M, Kobayashi Y, Yasutake T, Okada M, Okita K, ASCITES Clinical Pharmacology Group. (2012). The pharmacokinetics and pharmacodynamics of tolvaptan in patients with liver cirrhosis with insufficient response to conventional diuretics: a multicentre, double-blind, parallel-group, phase III study. J Int Med Res.

